# Effectiveness of the Beat the Kick intervention in addressing substance abuse among adults with mild intellectual disabilities in the Netherlands: study protocol for an open-label, multicenter, superiority randomized controlled trial

**DOI:** 10.1186/s13063-025-09299-3

**Published:** 2025-11-27

**Authors:** Rosemarie Gideonse, Noud Frielink, Carlo Schuengel, Joanneke E.L. van der Nagel, Petri J.C.M. Embregts

**Affiliations:** 1 Academic Collaborative Center for Living With an Intellectual Disability, Department of Tranzo, Tilburg School of Social and Behavioral Sciences, Tilburg University, Tilburg, The Netherlands; 2 Department of Clinical Child and Family Studies, VU Amsterdam, Amsterdam, The Netherlands; 3 Nijmegen Institute for Scientist-Practitioners in Addiction (NISPA), Nijmegen, The Netherlands; 4 Department of Human Media Interaction (HMI), University of Twente, Enschede, The Netherlands; 5 Tactus, Center for Addiction and Intellectual Disability, Deventer, The Netherlands; 6 Aveleijn, Borne, The Netherlands

**Keywords:** Beat the Kick intervention, Mild intellectual disability, Substance abuse, Randomized controlled trial, RCT, Study protocol, Care-as-usual

## Abstract

**Background:**

People with mild intellectual disabilities (MID) are at elevated risk of substance-use problems. Beat the Kick is a motivation-focused, MID-adapted pre-treatment program. This trial aims to test whether Beat the Kick increases autonomous motivation to enter substance-use treatment versus care-as-usual (CAU), and to examine effects on substance use, satisfaction of basic psychological needs, treatment engagement, and acceptability.

**Methods:**

Open-label, multicenter superiority randomized controlled trial in the Netherlands. We will recruit 138 adults (≥ 18 years) with MID or borderline intellectual functioning (intelligence quotient (IQ) 50–85 with adaptive limitations) and hazardous substance use (Alcohol Use Disorders Identification Test (AUDIT) ≤ 19 or Drug Use Disorders Identification Test (DUDIT) ≤ 24; ≥ 12 months) from six intellectual-disability care organizations. Recruitment will be conducted jointly by researchers from Tilburg University and care professionals at the participating organizations. In a Zelen pre-randomization design, eligible clients will be randomized 1:1 to Beat the Kick or CAU using a centralized computer-generated sequence with variable block sizes, stratified by addiction type (alcohol vs cannabis/other drugs). Tilburg researchers will obtain informed consent after allocation (intervention: consent for intervention + assessments; CAU: consent for assessments only). As an open-label trial, only the statistical analyst will remain blinded to allocation via A/B-coded datasets; the allocation key is held by an independent coordinator until primary analyses are complete. Assessments occur at T1 (pre), T2 (post), T3 (1 month), and T4 (6 months). Safety is monitored at T2–T4 through systematic adverse event (AE) and serious adverse event (SAE) recording, and serious events are reported to the Ethics Review Board within 24 h.Primary outcome: autonomous motivation (Treatment Self-Regulation Questionnaire; 15 items; 1–5 scale). Secondary outcomes: substance use ( Substance Use and Misuse in Intellectual Disability Questionnaire (SumID-Q) with AUDIT (0–40; ≥ 8 hazardous; ≥ 20 probable dependence) and DUDIT (0–44; ≥ 6 men/ ≥ 2 women hazardous; ≥ 25 probable dependence)), satisfaction and frustration of psychological needs (Basic Psychological Need Satisfaction and Frustration Scale – Intellectual Disability Version (BPNSFS-ID); 24 items; 1–5), treatment engagement (yes/no), and participant satisfaction. Primary analyses will use intention-to-treat linear mixed-effects models with fixed effects for group, time, and group × time, adjusting for the stratification variable.

**Discussion:**

This study evaluates a motivation-focused, MID-adapted program under routine conditions. Anticipated challenges (open-label bias, retention) are addressed through exclusive trainer allocation per arm, supportive scheduling, and caregiver involvement. If effective, Beat the Kick could be integrated into standard practice to improve readiness for treatment, substance-use outcomes, and overall well-being in adults with MID.

**Trial registration:**

ISRCTN Registry, ISRCTN12571979. Registered on 11 June 2025. This trial was prospectively registered.

**Supplementary Information:**

The online version contains supplementary material available at 10.1186/s13063-025-09299-3.

## Administrative information

Note: the numbers in curly brackets in this protocol refer to SPIRIT checklist item numbers. The order of the items has been modified to group similar items (see http://www.equator-network.org/reporting-guidelines/spirit-2013-statement-defining-standard-protocol-items-for-clinical-trials/).

**Table:** 

Title {1}	Effectiveness of the Beat the Kick intervention in addressing substance abuse among adults with mild intellectual disabilities: study protocol for a randomized controlled trial
Trial registration {2a and 2b}.	ISRCTN Registry, ISRCTN12571979 Registered 11 June 2025. https://www.isrctn.com/ISRCTN12571979
Protocol version {3}	11 June 2025, version 1.0
Funding {4}	The Netherlands Organisation for Health Research and Development (ZonMW) funds this study through a research grant (08450412310007)
Author details {5a}	R. Gideonse, Tranzo, Tilburg University N. Frielink, Tranzo, Tilburg University C. Schuengel, VU Amsterdam J.E.L. van der Nagel, Institute for Scientist-Practitioners in Addiction (NISPA),The Netherlands, University of Twente, Tactus and Aveleijn P.J.C.M. Embregts, Tranzo, Tilburg University
Name and contact information for the trial sponsor {5b}	Tilburg University Social and Behavioral Sciences Tranzo Professor Cobbenhagenlaan 125 5037 DB Tilburg
Role of sponsor {5c}	Tilburg University, through Tranzo, is the sponsor and responsible for the study design, data collection, data management, data analysis, interpretation of the data, and the writing and submission of reports for publication. The funder (ZonMw) monitors the project through yearly reports and evaluations. The funder has no role in the study design and collection as well as the analysis and interpretation of the data.

## Introduction

### Background and rationale {6a}

Substance abuse, defined as the harmful or hazardous use of psychoactive substances, including alcohol, tobacco, and cannabis, poses a significant challenge among people with mild intellectual disabilities (MID). In the Netherlands, a large study among 419 adults with MID/borderline intellectual functioning (BIF) receiving support from 16 disability services reported 64% current alcohol use, 62% smoking tobacco, and 15% cannabis or other illicit drug use (data collected 2010–2011) [1]. A 2019 systematic review (138 studies; 2000–2018) of international research on substance use and substance use disorders (SUD) in individuals with MID/BIF concluded that this group has elevated risks across diverse settings; for example, North American population-based data indicate SUD prevalences of roughly 6% in adults with intellectual disabilities versus ~4% in those without, while UK community cohorts reported about 1% diagnosed SUD in mixed-severity ID, with higher rates in clinical and forensic samples [2]. Consistent with this, a nationwide Swedish cohort using matched population and sibling comparisons found that individuals with MID had an increased risk of any substance use–related problem versus the general population (HR 1.82, 95% CI 1.73–1.91), which remained significant though attenuated in sibling analyses (HR 1.37, 95% CI 1.23–1.51) [3]. Together, these findings highlight both the national and international scope of the problem.

MID is characterized by limitations in intellectual functioning and adaptive behavior with onset during the developmental period; the mild range corresponds roughly to intelligence quotient (IQ) 50–55 to 70–75 [4]. In Dutch practice and service planning, individuals with borderline intellectual functioning (BIF; IQ 70–85) are often grouped with MID because they share many characteristics and face similar challenges in SUD care [5]. Reflecting this inclusive approach, this study first presents results for individuals with BIF; thereafter, “MID” is used to refer to both MID and BIF.

#### Barriers to treatment for individuals with MID

People with MID encounter significant barriers to addiction treatment, as services often fail to address their specific cognitive, emotional, and motivational needs. This contributes to health disparities, with unmet healthcare needs and limited access to effective interventions remaining common. For instance, mainstream addiction programs in the United States frequently exclude people with MID due to challenges in adapting to their needs [6]. In Canada, people with MID face considerable barriers in healthcare access, as general medical services often lack provider training, adapted communication strategies, and systematic support for their complex needs [7]. In Sweden, people with MID have been found to have an 81% higher risk of substance-related problems, particularly those with psychiatric comorbidities. Despite a well-developed healthcare system, inadequate tailored interventions and poor service coordination limit access to effective addiction treatment.

In the Netherlands, a significant treatment gap for people with MID and addiction was identified over a decade ago. A study using capture-recapture methodology showed that a substantial number of individuals with co-occurring MID and substance abuse disorders remained unidentified and unsupported by either care system [8]. Their findings highlighted how uncoordinated care between intellectual disability facilities and substance abuse treatment centers left many individuals undetected and unsupported by either system. Since then, several specialized treatment protocols and interdisciplinary teams have been developed to bridge this gap, including tailored screening tools, adapted cognitive-behavioral approaches, and initiatives aimed at improving collaboration between care providers. However, despite these advancements, such protocols have not been implemented consistently across services and regions. As a result, substantial disparities in access to coordinated and appropriate care remain, and the group previously shown to fall through the cracks of both systems continues to face exclusion risks [8].

Broader healthcare system issues exacerbate these challenges. Doherty et al. [9] identified several systemic barriers in primary care, including inadequate provider training, poor communication, and limited involvement of people with MID in decision-making. These barriers contribute to fragmented care and exclusion from essential services. Addressing these issues requires tailored addiction programs alongside systemic reforms, such as enhanced provider training, accessible communication strategies, and collaborative decision-making. Among the various barriers, low treatment motivation emerges as a particularly pivotal and modifiable factor. Research has shown that individuals with MID often experience limited insight into their substance use, struggle to articulate personal goals, and have difficulties initiating and sustaining treatment engagement [10, 11]. Motivation is a key predictor of treatment adherence and success across populations [12], and in the MID population it is closely linked to the quality of relational support and the extent to which autonomy, competence, and relatedness are supported [13]. However, most substance abuse services are not designed to foster these conditions.

As such, motivational barriers are not simply part of a broader problem—they are a core obstacle to effective care, and one that must be addressed first in order to enable access and engagement with treatment. This forms the rationale for the Beat the Kick intervention, which explicitly targets motivation as the primary outcome. Beat the Kick aims to strengthen autonomous motivation using approaches grounded in motivational interviewing and self-determination theory. By addressing this critical barrier, Beat the Kick seeks to create the conditions under which other forms of support can become accessible and effective.

#### Direct consequences of substance abuse

Substance abuse exacerbates vulnerabilities among people with MID, worsening mental and physical health and increasing social isolation. Cognitive limitations, such as difficulties in self-regulation and decision-making, can increase reliance on maladaptive coping (including substance use) [1]. These challenges compound common comorbidities such as anxiety and depression, creating barriers to effective care [2]. Evidence from a Swedish nationwide population-based cohort—linking national healthcare and population registers—shows that people with MID have a higher risk of any substance-use–related problem than the general population (HR 1.82, 95% CI 1.73–1.91), which remains elevated in sibling analyses (HR 1.37, 95% CI 1.23–1.51); risks are especially high with psychiatric comorbidity (e.g., bipolar disorder HR ≈ 8.22; psychotic disorder HR ≈ 6.52) [4]. In addition, studies in MID/BIF indicate that underlying motives (e.g., emotion regulation, social approval) are closely tied to the severity and frequency of use, underscoring the need for interventions that target these drivers [14].

#### Indirect consequences for caregivers and support networks

Substance abuse among people with MID also impacts caregivers and support networks, often leading to emotional exhaustion and strained relationships. Caregivers of individuals with MID and comorbid psychiatric disorders have been shown to experience significantly higher levels of stress and psychological distress, largely due to the complexity of the care required [15]. This underscores the importance of providing caregivers with specialized support and training. When substance abuse becomes more severe, caregivers and support networks often face additional burdens, as they must manage escalating behavioral and emotional challenges. This highlights the need for targeted, personalized interventions that not only address substance abuse itself but also support the broader network surrounding the individual.

#### Current therapeutic approaches

Evidence-based approaches such as cognitive behavior therapy (CBT) have demonstrated efficacy in addressing substance use disorders in people with MID when appropriately adapted [16]. Key adaptations include simplified language, visual aids, and concrete examples to enhance accessibility [17]. Motivational Interviewing (MI), which emphasizes intrinsic motivation through a person-centered approach, has also proven effective in fostering autonomy and sustainable behavioral change [18]. Research has shown that MI is applicable to individuals with MID, provided that techniques are tailored to their language level, cognitive abilities, and tendency toward socially desirable responses [13]. Additionally, certain professional characteristics—such as trustworthiness, engagement, and empathy—are essential for optimizing treatment motivation and outcomes.

CBT interventions have shown promise in improving mental health outcomes for people with MID, though more robust, long-term trials are needed to confirm these findings [19]. While much of the existing research focuses on group-based CBT formats, it is also relevant to examine whether CBT-based approaches that incorporate motivational interviewing, such as Beat the Kick, are effective in the Dutch context. This combination targets emotional regulation and self-awareness while enhancing treatment engagement and outcomes [20, 21].

#### Development and evidence supporting Beat the Kick

The Dutch Beat the Kick intervention is a specialized pre-treatment program designed to meet the cognitive, emotional, and motivational needs of individuals with intellectual disabilities (Table 1). It integrates CBT and MI to build foundational skills and enhance motivation, addressing key barriers to treatment engagement and effectiveness (22, 23). Adapted CBT approaches are particularly effective in managing co-occurring conditions like anxiety, which often exacerbate substance use issues [22].

**Table 1:** Beat the Kick intervention

Beat the Kick intervention	Time (minutes)	Attendees
Recommendation:		
Intake with administration of SumID-Q	60 min	
Beat the Kick sessions	45–60 min	Trainer and participant
• 1: The first acquaintance		
• 2: Focusing on the participant’s use		
• 3: When are you addicted?		
• 4: When do you get urges?		
• 5: Urges: How do they work?		
• 6: The 3Cs: Catching, checking and changing		
• 7: What to do when you get the urge?		
• 8: Genogram		
• 9: Take Care card		
• 10: Closing session		

An initial evaluation using a single-case experimental design (SCED) demonstrated the promise of Beat the Kick in improving outcomes for people with intellectual disabilities [21]. However, while SCEDs offer strong internal validity, they are limited in generalizability, necessitating complementary randomized controlled trials (RCTs) to test efficacy in more representative samples (Bhatt and Gentile, 2021).

#### Global potential and future research

Beat the Kick addresses a critical gap in addiction treatment for people with MID. While countries such as Canada, the UK, and the USA recognize the need for similar interventions, progress is hindered by a lack of evidence-based models [6]. Large-scale RCTs could rigorously test the program’s efficacy and establish it as a structured, evidence-based pre-treatment approach within addiction care.

#### Proposed effectiveness study

A randomized controlled trial will evaluate the effectiveness of Beat the Kick in enhancing motivation for treatment, improving substance use outcomes, and increasing mental health and treatment engagement compared to standard addiction treatments. Insights from this study will inform best practices for addiction treatment and contribute to reducing health disparities for people with MID globally.

### Objectives {7}

#### Primary objective

The primary objective of this RCT is to evaluate the effectiveness of the Beat the Kick intervention compared to care-as-usual (CAU) in adults with MID, focusing on enhancing autonomous motivation for substance abuse treatment. The central hypothesis is that individuals receiving the Beat the Kick intervention will show greater improvement in autonomous motivation for engaging in substance abuse treatment from pre-test to post-test compared to those in the CAU group.

#### Secondary objective

The secondary objective is to assess the effectiveness of Beat the Kick in reducing substance use. This will be measured by comparing changes in substance abuse from pre-treatment to follow-up between the experimental and CAU groups. The hypothesis is that participants in the experimental group will show a greater reduction in substance use compared to those in CAU at follow-up.

### Trial design {8}

This multicenter, parallel-group superiority randomized controlled trial employs a Zelen design to evaluate the effectiveness of the Beat the Kick intervention in enhancing autonomous motivation and reducing substance use among adults with MID. The Zelen design ensures unbiased group assignment by randomizing 138 consecutive participants into two groups—treatment and CAU—prior to obtaining informed consent, thereby minimizing selection bias and mirroring real-world clinical scenarios [23]. Participants are allocated in a 1:1 ratio, with 69 individuals in each group.

Randomization occurs at the individual level, which introduces the potential risk of carry-over effects if therapists treat participants in both the CAU and Beat the Kick intervention groups. To mitigate this risk, therapists are exclusively assigned to either the intervention or CAU group, ensuring no overlap in their caseloads. Additionally, therapists from the intervention and CAU groups are employed by different organizations and do not collaborate or exchange information during the study period. This separation prevents contamination and enhances the validity of the findings. Following the study, therapists can provide valuable reflections on their experiences with the training, including its perceived effectiveness and practical applicability, offering further contextualization of the outcomes.

Assessments are conducted at four time points: pre-treatment (T1), post-treatment (T2), and follow-up evaluations at 1 month (T3) and 6 months (T4) after the intervention. This schedule allows for the measurement of both immediate and sustained effects of the intervention.

## Methods

### Study setting {9}

**Fig. 1:**
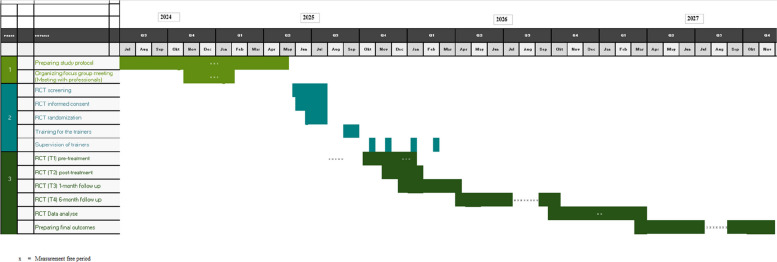
Gantt chart

This multicenter study will be conducted in six healthcare organizations for people with intellectual disabilities in the Netherlands, all partners of the Academic Collaborative Center Living with an intellectual disability (Tranzo, Tilburg University). Each participating organization cares for approximately 20 adults per year with co-occurring MID/BIF and hazardous substance use who match our eligibility profile. To secure a sufficient recruitment pool and geographic spread, six organizations are included (combined projected pool ≈120/year), supporting the feasibility of recruiting *N* = 138 within the planned accrual period. The organizations are distributed across urban and rural regions, providing a heterogeneous and therefore representative sample of adults with MID in Dutch intellectual disability services (Fig. 1).

### Eligibility criteria {10}

#### Inclusion criteria

Participants are eligible if they meet the following criteria:

- MID or BIF: Defined by an IQ of 50–70 (MID) or 70–85 (BIF) combined with severe adaptive functioning limitations (defined by significant impairments in at least two of the following domains: conceptual, social, and practical skills, as outlined in the DSM-5). Both groups are collectively referred to as “individuals with MID,” reflecting their shared characteristics and support needs. In the Netherlands, these groups qualify for specialized mental health care and are often grouped in research, practice, and policy contexts [5].
- Substance abuse: Hazardous alcohol or drug use that negatively impacts physical, psychological, interpersonal, or social well-being for a duration of at least 12 months [10]. To focus the intervention on individuals with problematic substance abuse rather than severe substance use disorders, participants with indications of high-intensity or clinically complex use patterns will be excluded. This will be assessed using the Substance Use and Misuse in Intellectual Disability Questionnaire [24], which includes the Alcohol Use Disorders Identification Test (AUDIT) and the Drug Use Disorders Identification Test (DUDIT) to screen for substance abuse severity. Participants who exceed the established clinical thresholds for high-risk use (AUDIT > 19 or DUDIT > 24) will not be eligible to participate, as their level of use suggests the need for more intensive, specialized addiction care [24, 25].
- Lack of autonomous motivation: Participants must exhibit low autonomous motivation to change their substance use, as this is the primary target of the intervention. Autonomous motivation will be measured using the Treatment Self-Regulation Questionnaire (TSRQ) [21], which assesses the extent to which individuals engage in change based on personal value, interest, or internal goals [26]. This criterion ensures that the intervention is tested in individuals who are not yet ready or internally motivated to change, in line with the study’s aims.
- Age: Participants must be 18 years or older to ensure legal competence for informed consent and appropriateness of intervention content.
- Language proficiency: Participants must have sufficient Dutch language proficiency (minimum B1 level) to understand the intervention content, complete study measures, and engage in therapeutic conversations. This ensures the validity of assessments and meaningful participation.

#### Exclusion criteria

Participants will be excluded if any of the following apply:

- Medication-related substance use: Individuals whose substance use is exclusively related to prescribed medications with potential for misuse (e.g., opioids, benzodiazepines, or ADHD medications like methylphenidate) will be excluded. This distinction is made in order to focus the intervention on non-medical substance misuse and to avoid medical confounds [2].
- Lack of response or confusion: Individuals who are unable to provide meaningful verbal or non-verbal responses or who display significant confusion will be excluded. This ensures participants can engage with intervention content and assessments in a reliable and ethical manner.
- Concurrent or planned substance use treatment: Individuals currently receiving, or planning to begin, other substance use interventions during the study period will be excluded to prevent treatment contamination and ensure internal validity of outcomes [27, 28].
- Unstable living situation: Homelessness or unstable housing will be grounds for exclusion due to the increased risk of dropout, relapse, and inability to guarantee follow-up. Stable living conditions are a known prerequisite for effective outpatient interventions (29, 30]).
- Pregnancy: Pregnant individuals will be excluded due to ethical concerns and potential medical complications related to withdrawal, stress, and fetal risk [31].
- Inability to give informed consent: Individuals who are legally restricted from consenting or who demonstrate cognitive impairments beyond the MID/BIF range will be excluded. However, when applicable, legal representatives may provide consent on their behalf in accordance with ethical guidelines, allowing participation when appropriate [32]. This ensures ethical standards are upheld while maximizing inclusion.

#### Patient and public involvement

People with lived experience of MID and substance abuse were involved in the developmental phase of Beat the Kick. They contributed feedback on intervention materials (e.g., visual aids, examples) and pilot-tested sessions to ensure accessibility. They will also be involved in dissemination activities, including co-creating accessible summaries and video materials.

### Who will obtain informed consent? {26a}

Recruitment will be conducted jointly by researchers from Tilburg University and care professionals at the participating organizations: care professionals identify and pre-screen eligible clients and introduce the study; Tilburg University researchers then confirm eligibility, provide standardized information in accessible formats, and obtain written informed consent. Researchers from Tilburg University with expertise in intellectual disabilities and substance abuse will be responsible for obtaining informed consent. They will ensure that all participants, or their legal representatives where applicable, fully understand the nature of the study, the intervention, and any potential risks or benefits. This process will be guided by the Zelen design, which facilitates ethical and transparent participant engagement.

Participants randomized to the intervention group (Beat the Kick) will be approached after randomization. Researchers will provide a detailed explanation of the intervention and study procedures, seeking their consent to participate in both the intervention and the study assessments, such as questionnaires and interviews. Participants randomized to the CAU group will be informed of their allocation and asked to consent to participate in the study assessments only, which are essential for evaluating study outcomes but do not involve active participation in the intervention. The primary goal is to seek consent directly from participants whenever possible. In cases where a legal representative is required, formal consent will also be obtained from the representative, in compliance with Dutch law and ethical guidelines. Consent from both the participant and their representative will be required, as the participant’s engagement is crucial for a motivation-based intervention. Researchers will use tailored communication strategies, including clear language and visual aids, to address any questions or concerns and ensure participants’ comprehension of the study details.

All questionnaires will be completed digitally or on paper, with assistance from the researcher if needed, ensuring accessibility and efficiency in data collection. Model versions of the participant information sheet and consent form for both study groups are provided as supplementary materials (Additional files 1 and 2).

### Additional consent provisions for collection and use of participant data and biological specimens {26b}

There are no plans for the collection of biological specimens in this study. However, participants will be informed about the potential use of their data in future ancillary studies and will have the option to provide separate consent for this purpose. The consent forms will clearly outline data confidentiality, usage, and the right to withdraw consent for future data use at any time, without affecting their participation in the main study. This approach, approved by the Ethics Review Board of Tilburg University (RP 1983), ensures that participants or their legal representatives can make informed decisions regarding the use of their data. Additionally, any future research utilizing this data will require approval from an independent ethics committee to ensure adherence to ethical standards and the protection of participants’ rights and privacy.

## Intervention

### Explanation for the choice of comparators {6b}

The comparator in this study is the CAU condition. This choice enables a direct evaluation of the effectiveness of the Beat the Kick intervention relative to the standard care typically provided to people with MID and substance abuse problems. Participants in the CAU group will receive support from their intellectual disability healthcare organization, which may involve a multidisciplinary team including physicians, nurses, psychologists, social workers, and counselors.

Standard care in the CAU group encompasses a wide range of approaches tailored to individual needs. These may involve techniques to enhance readiness for change, as well as strategies focused on building supportive relationships between the individual and their caregiver. The aim is to create a stable and safe environment in which individuals feel valued and engaged in meaningful daily activities that foster a sense of competence and reduce challenging behaviors. Standard care may also include guidance, information, and assistance related to substance use and intellectual disabilities, along with regular check-ups to monitor progress, assess needs, and provide general support. Participants in the CAU group will not receive the structured Beat the Kick intervention. However, they will continue to receive care from their regular healthcare providers, including support related to substance use and intellectual disabilities. This ensures that all participants receive an ethically appropriate level of baseline care while allowing for a valid comparison with the added value of the intervention.

In addition, the use of a CAU comparator rather than a waitlist control helps prevent bias that could arise from nocebo effects. Participants placed on a waitlist may experience demoralization, reduced motivation, or worsening of symptoms due to the absence of support, which can artificially inflate the intervention’s observed effectiveness. By comparing Beat the Kick to the care that is actually available in practice, the study maintains both clinical relevance and methodological integrity.

### Intervention description {11a}

The Beat the Kick intervention consists of ten weekly individualized sessions, each lasting 45–60 min, designed to help participants understand and change their substance abuse patterns, identify triggers, and adopt healthier coping strategies. The intervention draws upon Self-Determination Theory [26] and Motivational Interviewing [33] to foster autonomous motivation. It does so by supporting the basic psychological needs of autonomy, competence, and relatedness, and by creating a safe, supportive environment in which participants are encouraged to explore and commit to meaningful behavior change.

Each participant is guided by a healthcare professional who has completed the “Train the Trainers: Beat the Kick” program. The program begins with an introductory session in which the trainer builds rapport, discusses expectations, and tailors the intervention to the participant’s specific needs and circumstances. To prevent potential carry-over effects, staff members supporting participants in the intervention group will not simultaneously support participants in the CAU group. This separation ensures that intervention-specific knowledge and techniques are not inadvertently applied in the control condition, preserving the integrity of the comparison.

Subsequent sessions are structured thematically. The second and third sessions help participants explore their substance use behaviors and attitudes through interactive activities such as online assignments, video clips, and discussions. Concepts like the distinction between substance addiction and substance abuse are clarified with visual aids. In sessions four through six, cognitive-behavioral techniques are introduced using the ABC model [34], which links triggers, beliefs, and behaviors. These sessions focus on identifying craving triggers and developing healthier responses.

Session seven emphasizes identifying personal values and interests to explore alternative activities that can replace substance use, while session eight focuses on evaluating the participant’s social network and identifying individuals who can offer support during challenging moments. The final two sessions consolidate the learnings by developing a personalized action plan and discussing options for ongoing support. The program concludes with a certificate recognizing participants’ efforts.

Before the intervention starts, participants’ regular support staff attend a 1.5-h clinical briefing session delivered by the trainer. This session is designed to improve staff members’ understanding of substance use and the intervention content, enhancing their ability to support participants appropriately. Additionally, a helper from the participant’s own social network provides ongoing support throughout the intervention period, offering a trusted and accessible source of encouragement and reflection.

### Criteria for discontinuing or modifying allocated interventions {11b}

Participants may discontinue the intervention for various reasons. They are free to withdraw from the study at any time without providing an explanation, and their autonomy in making this decision will be fully respected. If a participant’s condition deteriorates, for example due to increased substance use, the onset of severe psychiatric symptoms, or emerging safety concerns, healthcare professionals may advise discontinuation of the intervention in order to safeguard the participant’s well-being.

Discontinuation may also be considered if the participant experiences substantial emotional distress or other adverse effects that appear to be directly related to the intervention. In situations where participants consistently miss sessions or have difficulty engaging with the intervention, additional support will be offered to promote continued involvement. Whenever possible, modifications will be made in collaboration with the participant and their caregiver to enhance engagement, rather than opting for discontinuation. All forms of discontinuation, including partial disengagement such as repeated absences or reduced participation, will be documented and may be analyzed as outcomes in their own right. These instances may provide valuable insight into motivational dynamics or barriers to sustained participation.

If a participant voluntarily initiates formal addiction treatment during the study period, this will be recorded as a meaningful outcome rather than treated as a reason for discontinuation. This step will be interpreted as an indication of increased autonomous motivation to change, provided the decision to seek treatment is made voluntarily and not due to external pressure.

### Strategies to improve adherence to interventions {11c}

Several strategies will be employed to promote adherence to the intervention. A coordinated team, including trainers, supervisors (such as senior practitioners or clinical leads overseeing intervention fidelity), and the primary researcher as the independent monitor, will work together to ensure fidelity and quality. Trainers will be carefully selected from organizations familiar with the intervention’s goals and context. They will meet specific qualifications, including expertise in intellectual disabilities and addiction and, ideally, knowledge of MI. These trainers will receive formal training through RINO Zuid, a Dutch institute specializing in mental health and social care professional development, which includes theoretical instruction, practical exercises, and ongoing supervision to ensure high-quality and consistent delivery of the intervention.

Supervisors will provide regular oversight and feedback to trainers, addressing challenges to maintain fidelity. The independent monitor (primary researcher) will periodically review intervention sessions by analyzing session recordings or attending selected sessions, ensuring adherence to protocols and providing objective feedback to trainers. Trainers will also remain available for participant questions and concerns between sessions, but no additional structured check-ins will be required to maintain feasibility. The intervention will be tailored to align with each participant’s personal goals and needs, ensuring relevance and sustained motivation.

Fidelity will be monitored through detailed documentation, including trainer-completed checklists after each session, and regular reviews by the monitoring team to assess adherence to established protocols. Discrepancies or concerns will be addressed promptly through research team meetings, enabling continuous improvement in adherence strategies.

### Relevant concomitant care permitted or prohibited during the trial {11d}

Participants will continue receiving standard care from their healthcare organizations, which may include psychosocial support, medical check-ups, and general counseling. If participants voluntarily initiate other structured substance use treatment programs during the study period, this will be documented as a meaningful outcome rather than considered a reason for discontinuation. Such a step will be interpreted as an indicator of increased autonomous motivation to change, provided it is initiated voluntarily and not under external pressure. This approach ensures that the study can accurately assess the specific impact of the Beat the Kick intervention on motivational and substance use outcomes.

Participants will not be permitted to participate in other research trials related to substance abuse treatment during the study period. All concurrent treatments and support services will be thoroughly documented and considered in the analysis to evaluate their potential influence on study outcomes.

### Provisions for post-trial care {30}

After the intervention, participants interested in additional support will be provided with information about follow-up options through their healthcare organizations. Trainers will work with participants to identify suitable follow-up options tailored to their needs, such as individual counseling or, where available, more intensive substance abuse treatment. Given existing treatment gaps and regional differences in service accessibility, efforts will be made to align follow-up advice with realistic options within participants’ local care networks. Any participant experiencing distress related to the intervention will receive appropriate psychological support, including counseling or referrals to specialized services.

### Outcomes {12}

The primary outcome is the change in autonomous motivation for substance-abuse treatment, measured using an adapted version of the TSRQ [21]. The TSRQ comprises 15 items rated on a 5-point Likert scale (1 = completely untrue; 5 = completely true). Subscales are scored as means (range 1–5) and include autonomous motivation (internal consistency α≈0.84; primary endpoint), controlled motivation (α≈0.78), and amotivation (α≈0.41; analyzed exploratorily due to lower reliability). As participation in the intervention and any subsequent treatment is voluntary, baseline motivation may be relatively high; this will be considered when interpreting change. Assessments will occur at pre-treatment (T1), post-treatment (T2), and follow-ups at 1 month (T3) and 6 months (T4), with the primary endpoint defined as Δ(T2–T1) on autonomous motivation and maintenance examined at T3/T4. To optimize comprehension for MID/BIF participants, all self-reports will be interviewer-assisted.

Secondary outcomes include reductions in substance use and related problems, measured with the SumID-Q (patterns, frequency, consequences), which embeds the AUDIT and DUDIT screens. The AUDIT has 10 items (item scores 0–4; total 0–40) with commonly used cut-offs ≥ 8 for hazardous use and ≥ 20 for probable dependence. The DUDIT has 11 items (item scores 0–4; total 0–44) with cut-offs for hazardous use of ≥ 6 for men and ≥ 2 for women, and ≥ 25 for probable dependence. Both continuous totals and categorical classifications (none/hazardous/probable dependence) will be analyzed at T1, T2, T3, and T4 (recall periods per instrument guidance). Psychological need satisfaction and frustration will be assessed with the Basic Psychological Needs Satisfaction and Frustration Scale – Intellectual Disabilities (BPNSFS-ID; 24 items, 5-point Likert), yielding mean scores (range 1–5) for autonomy, competence, and relatedness (higher satisfaction = greater fulfillment; higher frustration = greater need thwarting) at T1, T2, T3, and T4 [35].

In addition, treatment engagement will be captured as initiation of formal SUD treatment (yes/no)—defined as attending ≥ 1 treatment session or completing an intake—within the T3 window (± 1 month post-T2); follow-through on referrals (yes/no) will be recorded separately. Participant-reported acceptability will be assessed at T2 using brief Likert-type items (e.g., clarity, relevance, burden, usefulness, overall satisfaction; 1–5) and open-ended feedback. Safety will be monitored by systematic recording of intervention-related adverse events (AEs) and serious adverse events (SAEs) at T2, T3, and T4 via structured checklists and open queries; definitions, ascertainment procedures, and reporting timelines are detailed in Harms {23}.

### Participant timeline {13}

**Fig. 2:**
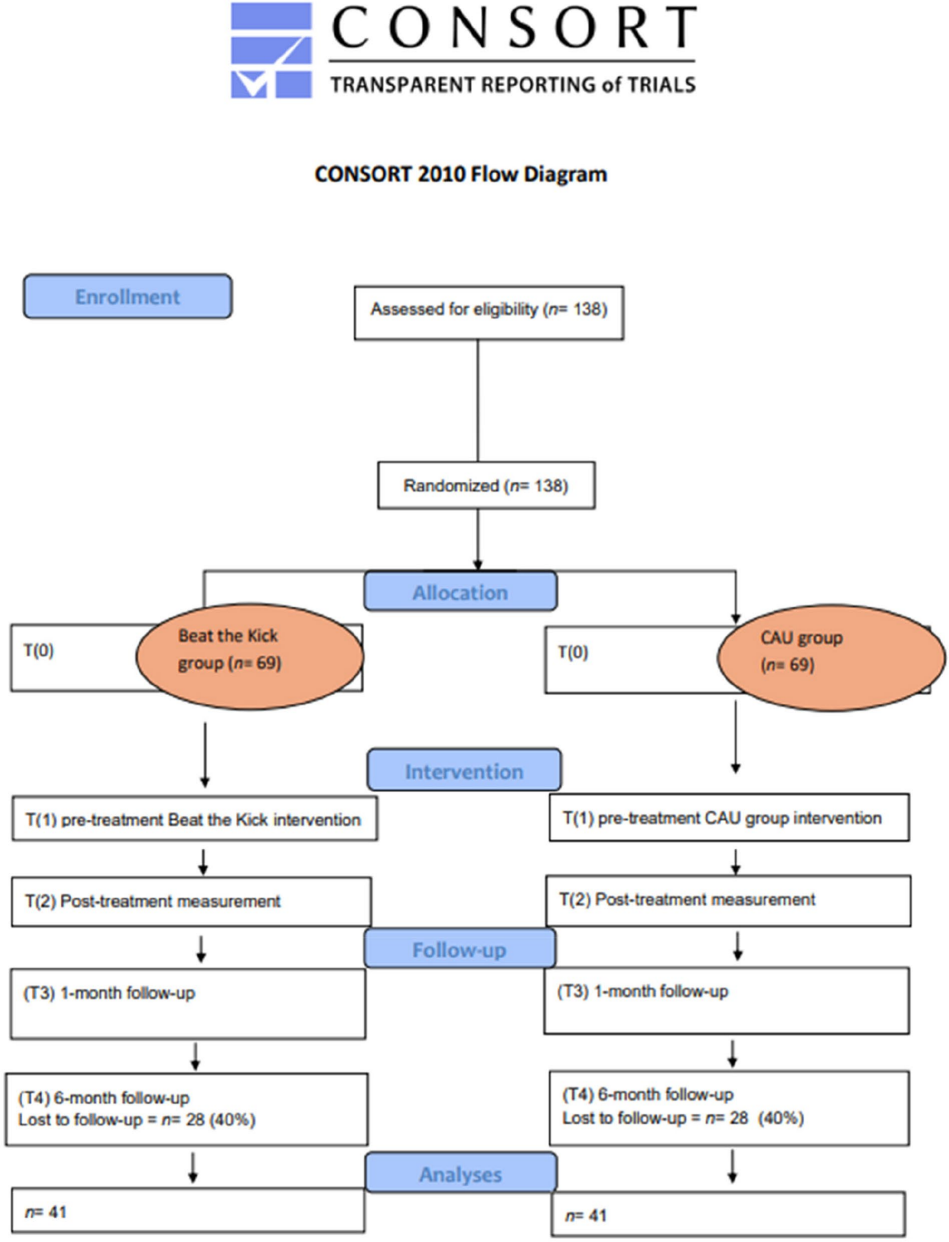
Consort diagram

**Fig. 3:**
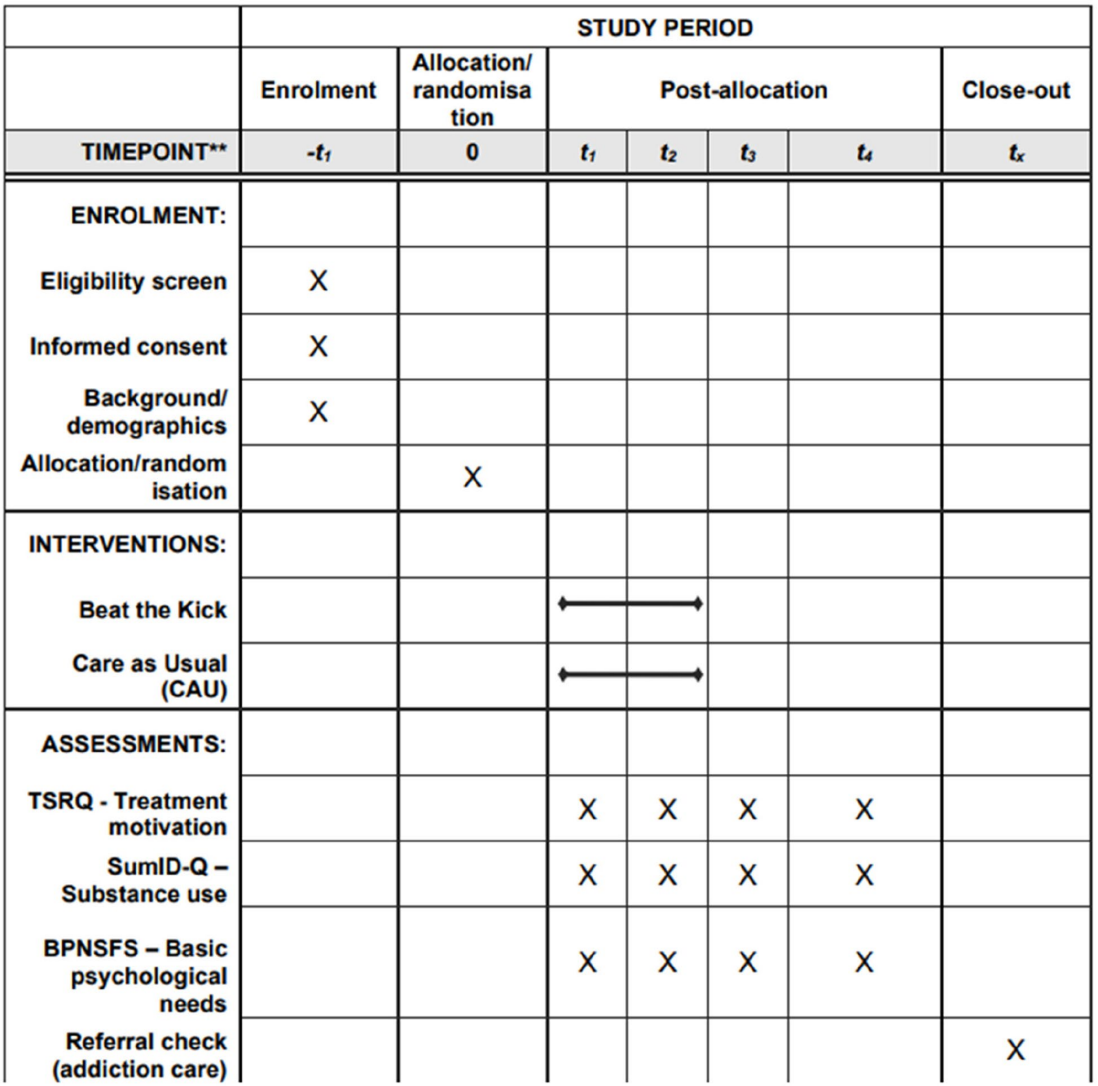
SPIRIT schedule of enrolment, interventions, and assessments

Participants will be enrolled in the study following a thorough screening and informed consent process. The intervention period will last 10 weeks, during which participants will receive either the Beat the Kick intervention or CAU support. Assessments will be conducted at four key time points: pre-treatment (T1), post-treatment (T2), and during follow-ups at 1 month (T3) and 6 months (T4). This structured timeline ensures a comprehensive evaluation of both the immediate and long-term effects of the intervention. A schematic diagram of participant flow is presented in Fig. 2, and the detailed schedule of enrolment, interventions, and assessments is shown in Fig. 3.

### Sample size {14}

A sample size power analysis was performed using G*Power 3.1.9.7 [36] to ensure the study’s feasibility within its constraints and the available population of individuals with mild intellectual disabilities (MID) who abuse substances. The calculation was based on the primary hypothesis, which posits that participants receiving the Beat the Kick intervention will show greater improvement in autonomous motivation compared to those in the control group. The analysis assumed a between-group design with four measurement points: pre-treatment (T1), post-treatment (T2), and follow-ups at 1 month (T3) and 6 months (T4). An effect size of *f* = 0.25 (medium effect), a power of 80%, and a significance level of 5% (two-sided) were used, with default settings for the correlation among repeated measures (0.5) and the non-sphericity correction (ε = 1, assuming sphericity).

The power analysis indicated that a sample of 82 participants is required to detect the expected effect with 80% power. Given the elevated risk of dropout among individuals with MID and co-occurring substance use—estimated at approximately 40% based on previous studies—the recruitment target was increased to 138 participants.

This adjusted target accounts for anticipated attrition and ensures that a sufficient number of participants will complete the study to maintain statistical power. Although recruiting 138 individuals from this population poses logistical challenges, it is considered feasible through targeted recruitment strategies, including partnerships with care providers and the use of established professional networks. This adjusted recruitment plan acknowledges the inherent risks of higher dropout rates but ensures the robustness of the study’s findings and feasibility within its framework.

### Recruitment {15}

Participants will be selected from the caseloads of six organizations affiliated with the Academic Collaborative Center Living with an intellectual disability (AWVB) at Tranzo, Tilburg University. Eligible individuals with MID who are not currently receiving substance abuse treatment will be identified through a random selection process carried out by the principal researcher. From this pool, 138 people with MID meeting the inclusion and exclusion criteria will be pre-assigned to either the experimental or control group using a Zelen design. The randomization process will be conducted by the principal researcher in an independent capacity to ensure objectivity and minimize bias. The intervention will be delivered by behavioral specialists from the six participating healthcare organizations to ensure standardized implementation across all sites.

## Assignment of interventions: allocation

### Sequence generation {16a}

The study uses a Zelen pre-randomization design. Participants are individually randomized 1:1 to Beat the Kick or care-as-usual (CAU) using a centralized, computer-generated sequence with permuted blocks of variable size, stratified by addiction type (alcohol vs. cannabis/other drugs) to maintain balance across strata. Randomization is not clustered; clients from the same team may be allocated to either arm.

To minimize provider contamination while preserving individual randomization, trainers are assigned exclusively to a single arm (intervention or CAU) and do not deliver care in the other arm during the trial. Trainer and site identifiers are recorded at allocation; trainer will be modeled as a random effect in mixed-effects analyses to account for provider-level clustering. Any unavoidable cross-coverage will be documented a priori and examined in sensitivity analyses.

### Concealment mechanism {16b}

To ensure allocation concealment, a centralized randomization system will be employed. The allocation sequence will be securely stored on a protected university drive, which is access-restricted and does not require an additional password. The sequence will be accessible only to the principal researcher at Tranzo, Tilburg University, who is responsible for participant recruitment, intervention coordination, and data management. Once participants have completed enrollment and pre-treatment assessments, the principal researcher will assign participants to their respective groups and relay this information to the intervention team. This procedure ensures that allocation knowledge does not influence participant recruitment, thereby minimizing selection bias.

### Implementation {16c}

The primary researcher (in the role of data manager) at Tranzo will generate the allocation sequence using a computer-based random number generator. The primary researcher will also handle participant enrollment and obtain informed consent.

Once baseline assessments are completed, participants will be assigned to either the Beat the Kick or CAU group according to the allocation sequence. The principal researcher will oversee this process to ensure strict adherence to the randomization protocol and accurate group assignments.

## Assignment of interventions: blinding

### Who will be blinded {17a}

Due to the use of a Zelen design, participants cannot be blinded to their group assignment, as they are informed of their allocation after randomization. To mitigate potential analysis bias, the principal researcher, who will conduct the statistical analyses, will remain blinded to group assignments. Randomization will be implemented via a centralized computer-generated sequence; the resulting allocation key will be generated and securely stored by a trial coordinator who is not involved in the statistical analyses. For the purpose of analysis, the dataset will include only coded group labels (e.g., “Group A” and “Group B”), without fields that could reveal allocation. The key linking these codes to the actual arms will remain inaccessible to the analyst until all primary analyses are complete. This approach preserves the integrity of the randomization process and ensures unbiased statistical conclusions. The Zelen design further reduces selection bias and enhances external validity by simulating real-world clinical scenarios [37].

### Procedure for unblinding if needed {17b}

As this study is open-label and only the data analysts are blinded, no unblinding procedures will be required.

## Measures

### Plans for assessment and collection of outcomes {18a}

The primary outcome of this study, autonomous motivation, will be assessed using an adapted version of the TSRQ specifically tailored for substance abuse [21]. The TSRQ comprises 15 items divided into four subscales: autonomous motivation (e.g., “I would change my use because I choose to”), controlled motivation (e.g., “I would change my use because others expect me to”), and amotivation (e.g., “I am not at all concerned about why I would change my use”). Participants will respond on a 5-point scale from “completely untrue” to “completely true.” Mean scores for each subscale will be calculated by summing the corresponding items and dividing by the number of items. Previous studies have demonstrated strong reliability for the TSRQ, with Cronbach’s alpha values exceeding 0.73 for most subscales; however, amotivation has a lower reliability (α = 0.41). Autonomous motivation is associated with positive health outcomes, whereas controlled motivation correlates with negative outcomes [26].

Psychological need satisfaction and frustration will be measured using the BPNSFS-ID [35], adapted from the original BPNSFS [38]. This scale evaluates satisfaction and frustration across three psychological needs: autonomy, relatedness, and competence, comprising 24 items (eight items per need). Responses are rated on a 5-point Likert scale from 1 (“completely untrue”) to 5 (“completely true”). The BPNSFS-ID demonstrates high internal consistency (overall Cronbach’s alpha = 0.92) and robust factorial structure, with subscale alphas ranging from 0.78 to 0.92 and test-retest reliability ranging from 0.68 to 0.83 over two weeks. This study aims to explore whether the intervention influences substance use by impacting the satisfaction or frustration of these needs, potentially elucidating mechanisms underlying the intervention’s effectiveness.

Substance use will be assessed using the Substance Use and Misuse in Intellectual Disability Questionnaire (SumID-Q) [23]. This structured instrument follows a stepwise approach: it first identifies substances familiar to the participant (e.g., tobacco, alcohol, cannabis), then explores knowledge, attitudes, and contextual influences, and finally addresses personal use in a non-threatening manner, supported by visual aids to enhance understanding. The SumID-Q is specifically designed for individuals with MID and BIF, using simplified language, clear sentence structures, and visual support. Psychometric studies [23, 39] demonstrate adequate internal consistency and test–retest reliability. The embedded AUDIT and DUDIT scales have also shown good reliability: Cronbach’s α = 0.70 and 0.86 [40]. These findings support the SumID-Q as a valid and accessible tool for assessing substance use in individuals with MID-BIF, minimizing bias and facilitating accurate reporting (Table 2).

**Table 2:** Measures

	***Measures***				***Measurement***			
	Measure	Items (−/+)	Respondent	Type	T(1)	T(2)	T(3)	T(4)
Motivation and psychological needs	TSRQ	15	Participant	Questionnaire	*	*	*	*
	BPNSFS-ID	24	Participant	Questionnaire	*	*	*	*
Substance use	SumID-Q (Base form)	30	Participant	Questionnaire	*	*	*	*
	SumID-Q (Alcohol module)	49–61	Participant	Questionnaire	*	*	*	*
	SumID-Q (Tobacco module)	39–51	Participant	Questionnaire	*	*	*	*
	SumID-Q (Cannabis module)	50–62	Participant	Questionnaire	*	*	*	*
Total items, participant		108–131			108–131	108–131	108–131	108–131

Treatment engagement, defined as whether a participant has initiated a formal treatment program following the intervention, will be recorded as a binary outcome (yes/no). This will be assessed at post-intervention follow-up, using a combination of self-report and verification through the referring care provider or treatment records. This outcome will serve as a behavioral indicator of the participant’s motivation to engage with care and will support the interpretation of the intervention’s practical impact. Pre-treatment assessments will also document the number of prior treatments participants have undergone. This variable will be analyzed to determine its impact on participants’ readiness and responsiveness to the intervention. Understanding participants’ treatment history will provide insights into how prior experiences influence the effectiveness of Beat the Kick and may help identify subgroups that benefit more from specific strategies.

All assessors will undergo rigorous training to ensure consistent administration of these questionnaires, led by the primary researcher. Data collection forms and instruments will be securely stored on a protected online platform accessible only to the research team.

### Plans to promote participant retention and complete follow-up {18b}

Participants will receive reminders for the different assessment points (post-treatment, 1-month, and 6-month follow-ups). These communications will focus solely on the data collection schedule, without providing additional details about the content of the intervention.

If participants discontinue the intervention but agree to remain in the study, follow-up data will still be collected to uphold the intention-to-treat analysis. The reasons for discontinuation and the nature of collected data will be documented to ensure transparency and accuracy in reporting.

### Data management {19}

Data entry, coding, security, and storage will adhere to strict protocols to ensure confidentiality and accuracy. Each participant will be assigned a unique three-digit key code (001–200), linked to personal information via a list accessible only to two designated researchers. This list will be destroyed at the study’s conclusion to safeguard privacy. Sensitive data will be stored on secure servers at Tilburg University, with anonymized datasets archived in Dataverse, the university’s centralized research data repository.

To ensure pseudonymization during data collection, participants will be assigned a unique code before completing questionnaires. Participants will only enter this code on their questionnaires, avoiding any personal identifiers. If researchers assist in completing the questionnaires, they will not record identifying details, linking responses solely to the assigned code. Digital surveys will be configured to prevent the collection of IP addresses or other metadata that could compromise pseudonymization. A separate logbook will securely store the key linking participant names to codes, which will be permanently deleted upon study completion. Researchers involved in data collection will receive training from the study coordinator to ensure compliance with these anonymization protocols. All pseudonymized data will be stored on secure servers at Tilburg University for a period of 15 years, in line with Dutch regulations, after which they will be destroyed.

A comprehensive logbook will document all data collection activities, including measurements, dates, and participant details. The data management plan will follow FAIR principles (Findable, Accessible, Interoperable, Reusable), ensuring future usability while adhering to international privacy regulations overseen by institutional privacy officers. Findings will be disseminated through open-access publications and relevant platforms.

### Confidentiality {27}

All research data will be securely stored in restricted-access facilities. Participant privacy will be upheld using unique key codes that are linked to personal information only via an encrypted list. Data will be pseudonymized to ensure separation from personal identifiers. Researchers assisting in questionnaire completion will follow strict pseudonymization procedures, ensuring that no names or direct identifiers are recorded with responses.

Data sharing with third parties will require a “Data Transfer Agreement” (DTA) approved by Tilburg University’s Ethics Review Board. Sensitive data will remain on secure university servers, with pseudonymized datasets archived for future research in compliance with FAIR principles. Study findings will be published in open-access journals and shared via organizational platforms..

### Plans for collection, laboratory evaluation, and storage of biological specimens for genetic or molecular analysis {33}

This study does not involve the collection or storage of biological specimens, as noted in the “ Additional consent provisions for collection and use of participant data and biological specimens {26b}” section).

## Statistical methods

### Statistical methods for primary and secondary outcomes {20a}

Data analysis will be conducted using R version 4.5.0 or later [41], following standard procedures for descriptive and inferential analyses.

The primary objective of this RCT is to assess the effectiveness of the Beat the Kick intervention compared to CAU in enhancing autonomous motivation for substance abuse treatment among adults with MID. It is hypothesized that participants in the Beat the Kick group will exhibit greater improvement in autonomous motivation from pre-test to post-test compared to the CAU group, with an anticipated medium effect size.

Linear mixed models (LMM) will be employed to test this primary hypothesis using the lme4 package in R [42]. LMMs are particularly suited for repeated measures data, accommodating both fixed and random effects [42]. Models will include fixed effects for group (Beat the Kick vs CAU), time (T1–T4), and the group × time interaction (primary effect of interest), with random intercepts and random slopes for time at the participant level to capture individual trajectories. The randomization stratification variable “addiction type” (alcohol vs cannabis/other drugs) will be included as a fixed effect in all models to adjust for baseline heterogeneity between strata; exploratory interaction terms with group and time (e.g., group × time × addiction type) will be examined to assess effect modification [43]. Baseline outcome values will serve as covariates to control for initial differences, mitigating regression to the mean [44].

Analyses will primarily adhere to the intention-to-treat (ITT) principle, preserving the integrity of randomization and providing a conservative estimate of the intervention’s effectiveness [45]. This approach ensures that all participants are analyzed in their originally assigned groups, irrespective of adherence or dropout. Complementary per-protocol (PP) analyses will also be conducted for participants who completed the intervention as intended, providing additional insights. Missing data will be handled using maximum likelihood estimation, a robust method that assumes data are missing at random, leveraging all available information without requiring imputation [46].

Potential confounders, such as living arrangements (e.g., 24-h supervised care, outpatient services, or home-based support) and prior substance use treatment exposure, will be incorporated as covariates. These factors are expected to influence engagement and outcomes by shaping daily routines, support systems, and environmental stability. Trainer effects, which may vary due to differences in style, experience, or engagement, will be modeled as a random effect to minimize potential bias. Additionally, to prevent carry-over effects, trainers will be exclusively assigned to the intervention group, ensuring that training specific to Beat the Kick does not unintentionally influence CAU.

The secondary objective is to evaluate the intervention’s effectiveness in reducing substance use among adults with MID. It is hypothesized that the experimental group will show a greater reduction in substance use levels compared to the CAU group at follow-up. LMMs for secondary outcomes will mirror the primary model, including addiction type as a fixed effect (with exploratory interactions), and will adjust for baseline substance use levels.

To accommodate the Zelen design’s pre-randomization process, analyses will carefully consider group allocation at the pre-randomization stage, ensuring that group comparisons accurately reflect the intervention’s true effect. Sensitivity analyses will assess the robustness of results, including tests with and without outliers. Missing data will be handled using full information maximum likelihood (FIML), a robust method that leverages all available data and is generally preferred regardless of the underlying missingness mechanism [47]. Although FIML does not eliminate all bias under not-missing-at-random (NMAR) conditions, it typically yields less biased results than listwise deletion. Conclusions drawn under NMAR will be interpreted with appropriate caution, and the plausibility of the missing at random (MAR) assumption will be evaluated in light of the observed data structure and auxiliary variables.

Descriptive statistics will summarize baseline characteristics and outcome variables, including autonomous motivation and substance use levels. The normality of continuous variables will be assessed using the Shapiro-Wilk test [48] and visual inspection of histograms and Q-Q plots. Rather than applying data transformations or switching to non-parametric tests that may reduce interpretability and statistical power, linear mixed models will be retained even when normality assumptions are violated. This decision is supported by recent simulation research demonstrating the robustness of linear models to non-normal data, particularly in the context of hypothesis testing [49]. To safeguard validity, diagnostic checks (e.g., Cook’s distance, leverage values) will be conducted to detect influential data points. Where necessary, sensitivity analyses will be performed with and without such points to assess their impact on the results.

To ensure reliability and validity, Cronbach’s alpha will assess the internal consistency of scales [50], while intraclass correlation coefficients (ICCs) will evaluate reliability across repeated measures [51]. The results section will detail the statistical techniques employed, including maximum likelihood estimation for incomplete data [52], and highlight how LMMs effectively capture fixed and random effects, providing insights into individual variability and longitudinal trends. This rigorous methodology supports robust and interpretable findings (Table 3).

**Table 3:** Statistical analysis

	**Measure**	**Type of data**	**Timepoints**	**Statistical analysis**	**Subjects**
Motivation and psychological needs	TSRQ	Continuous	4	Linear Mixed Models	ITT, PP
	BPNSFS-ID	Continuous	4	Linear Mixed Models	ITT, PP
Substance use	SumID-Q	Continuous	4	Linear Mixed Models	ITT, PP

*ITT*, intention-to-treat analysis, including all randomized participants in the analysis; *PP*, per-protocol analysis, focusing on participants who adhered strictly to the protocol

### Interim analyses {21b}

No interim analyses are planned for this study. Decisions regarding early trial termination will be based solely on safety considerations or clear evidence of harm or benefit, as determined by the ethics committee.

### Methods for additional analyses (e.g., subgroup analyses) {20b}

Subgroup analyses will evaluate the intervention’s effects based on participants’ living arrangements (e.g., 24-h supervised care, outpatient services, or home-based support) to determine whether the care context moderates effectiveness. To minimize contamination or carry-over effects, staff members supporting participants in the intervention group will not be assigned to clients in the CAU group. In cases where overlap is unavoidable, measures will be taken to monitor and control for potential contamination effects in the analyses.

Additional subgroup analyses will explore whether the intervention’s effectiveness differs between individuals with MID and those with BIF, acknowledging potential differences in cognitive, communicative, and contextual resources between these groups.

The analysis will also consider the impact of prior exposure to substance use treatment, including the number and type of previous treatments. Demographic factors such as gender and age will be evaluated for potential moderating effects. Furthermore, patterns of substance use (alcohol and cannabis) will be assessed to determine whether specific use profiles influence the intervention’s effectiveness. This comprehensive approach aims to provide deeper insights into the factors shaping the intervention’s impact, guiding adaptations for specific populations and care settings.

### Methods in analysis to handle protocol non-adherence and missing data {20c}

The ITT principle will guide the analysis, ensuring robustness and generalizability by including all participants in their originally assigned groups, regardless of adherence to the intervention protocol or study completion. This approach minimizes biases from deviations, dropouts, or non-compliance, enhancing real-world applicability [45].

Missing data will be addressed using maximum likelihood estimation within the LMM framework. This method leverages all available data points, preserving dataset integrity while accounting for randomness in missingness without requiring imputation [46]. Combining ITT with maximum likelihood estimation ensures a comprehensive and unbiased assessment of the intervention’s effects.

### Plans to give access to the full protocol, participant-level data, and statistical code {31c}

Upon publication of the main trial results, the full protocol will be publicly accessible. Participant-level data will remain pseudonymized and confidential, in compliance with privacy and data protection regulations. However, de-identified datasets suitable for secondary analysis may be shared via Dataverse, subject to ethical approval and data protection standards. The statistical code used in the analysis will be available upon request, subject to ethical review and compliance with data protection standards.

## Oversight and monitoring

### Composition of the coordinating center and trial steering committee {5d}

The coordinating center (Tranzo, Tilburg University) oversees overall study management and administration. Day-to-day trial operations (screening, recruitment, scheduling, data capture, and protocol adherence) are handled by the Trial Management Group (TMG: principal investigator [chair], trial coordinator, data manager, lead trainer, and site leads).

A Trial Steering Committee (TSC) provides strategic oversight and independent advice. Membership: principal investigator (chair), co-investigators not involved in daily operations, an independent senior researcher from Tilburg University (not otherwise involved in the trial), the trial statistician, and site representatives from participating services. A PPI advisor (lived experience of MID/substance use) may attend selected meetings to advise on participant-facing materials; they are non-voting and not involved in safety adjudication.

Meeting cadence: the TMG meets monthly; the TSC meets quarterly (or ad hoc in response to safety signals). The TSC reviews recruitment and retention, protocol adherence, data quality summaries, adverse event reports, and any proposed protocol amendments, and escalates material issues to the Ethics Review Board.

#### Risk measures and mitigation strategies

To ensure the trial’s integrity and participant safety, potential risks and corresponding mitigation strategies have been identified in Table 4. These measures address anticipated challenges, including dropout rates, adverse events, and data security, ensuring that risks are actively monitored and mitigated throughout the study.

**Table 4:** Risk measures

Risk	Likelihood	Impact	Mitigating measures
Higher dropout rate	Medium	Reduced sample size	Flexible scheduling, involvement of helpers, regular check-ins, providing rewards
Adverse events (e.g., distress)	Low-medium	Emotional discomfort or dropout	Clear protocols, trained trainers, support from care teams
Limited understanding	Low	Incomplete or skewed data	Adapted materials, visual aids, helper involvement
Privacy or data breaches	Low	Loss of trust or ethical violations	Data security, pseudonymization, GDPR compliance
Emotional burden of reflection	Medium	Emotional strain or disengagement	Supportive approaches like MI and CBT, structured breaks, empathetic facilitation

### Composition of the data monitoring committee, its role, and reporting structure {21a}

No independent Data Monitoring Committee (DMC) is convened for this study. Given the open-label, behavioral, non-invasive nature of the intervention and its classification as posing no more than minimal physical risk, a full DMC would be disproportionate. Instead, safety and data quality are overseen through a two-layer structure:

1. Internal monitoring (trial team).
  The Principal Investigator (PI) oversees routine safety and quality checks after each assessment wave (T2/T3/T4). The trial team monitors data entry, protocol adherence, retention, and protocol deviations. A priori pause/stop criteria are defined (see Harms {22}).
2. Independent safety auditing.
  Proportionate independent oversight is provided by an Independent Safety Auditor (ISA)—a senior researcher at Tilburg University who is not involved in trial design, recruitment, intervention delivery, data collection, randomization, or statistical analysis, and is not in the PI’s line management. The ISA is appointed before first enrolment; their identity and contact details are recorded in the Trial Master File and are available to the Ethics Review Board (ERB) upon request. Cadence: quarterly audits (or after ~25% of inclusions, whichever comes first). Access/scope: the ISA may access unblinded safety data (not efficacy) when required to assess relatedness/expectedness; source verification is done on site with pseudonymized records where feasible.

Checklist:

- Presence/completeness of informed-consent and eligibility documentation;
- Inclusion pace and dropout/retention;
- Completeness/consistency of research data;
- Protocol deviations and corrective actions;
- AEs and SAEs logs and reporting timelines.

#### Reporting structure

ISA audit memos are sent to the PI and archived in the Trial Master File; key safety findings are summarized to the sponsor and the ERB at least annually or ad hoc if a safety signal emerges. SAEs are reported to the ERB within 24 h. If predefined thresholds are crossed (e.g., ≥ 2 related SAEs or unexpected moderate-to-severe distress in > 10% of participants), the PI will pause recruitment, request an ERB review, and implement protocol modifications as needed.

#### Scope

No interim efficacy analyses are planned; oversight focuses on participant safety and data integrity. This monitoring plan is proportionate to the trial’s risk profile and complies with ERB oversight requirements (see also Harms {22} for definitions, ascertainment, and reporting).

### Adverse event reporting and harms {22}

Adverse events will be closely monitored throughout the study. Harms are defined as any adverse psychological, behavioral, or medical event related to participation, including (but not limited to) increased distress, relapse episodes, psychiatric crisis requiring intervention, or hospitalization.

All reported or observed adverse events will be documented in detail (event, onset/offset, severity, relatedness, expectedness, action taken, outcome), including an assessment of severity and potential relationship to the intervention by the primary researcher. Ascertainment will occur during each study contact (T2, T3, T4) via participant self-report and facilitator observation, with additional spontaneous reporting allowed at any time; relevant chart information will be reviewed where applicable. SAEs will be reported to the Ethics Review Board within 24 h. If necessary, protocol modifications will be implemented promptly to ensure participant safety.

### Frequency and plans for auditing {23}

Proportionate internal auditing will occur after the first 10 inclusions and then quarterly (or ~10% file sampling, whichever comes first). Independent ISA audits are conducted on the same cadence (or at ~25% inclusion intervals) using a predefined checklist. Audit memos are sent to the PI and archived in the Trial Master File; key safety findings are summarized to the sponsor and the ERB at least annually or ad hoc if a safety signal emerges. Pause/stop criteria are prespecified (see Harms {22}); SAEs are reported to the ERB within 24 h.

### Plans for communicating important protocol changes to relevant parties {25}

Any amendments to the protocol, such as changes in eligibility criteria or outcome measures, will be communicated to relevant stakeholders, including the ethics committee. These amendments will also be documented in the trial registry and disseminated via appropriate scientific channels, such as peer-reviewed journals, conference presentations, and research networks, to maintain transparency. Direct communication of protocol changes to participants will not be undertaken.

### Dissemination plans {31a}

Study results will be disseminated via peer-reviewed publications, conference presentations, and reports on organizational websites. Participants will receive a plain-language summary and infographic within 6 months after completion of the primary endpoint analysis; these materials will also be shared via AWVB channels and social media. Co-created short videos with experts by experience will enhance accessibility. To support knowledge transfer, findings will be integrated into MBO/HBO curricula through guest lectures, workshops, and case studies on motivation and substance use in MID. Results will be reported irrespective of direction or significance. A concise summary will be posted to the trial registry record in line with registry policy.

## Discussion

Substance abuse is a significant issue among individuals with MID, impacting their physical health, autonomy, and motivation for treatment [1, 2, 14]. Autonomous motivation is critical to the success of substance abuse interventions, but remains underexplored in this population. The Beat the Kick intervention was developed to fill this gap by fostering intrinsic motivation through a structured program that targets psychological needs and self-regulation [21, 26, 33]. By addressing low autonomous motivation—a key modifiable barrier to care—this intervention aims to increase treatment engagement and improve outcomes in a group that is often excluded from or underserved by mainstream addiction services [6–8].

This RCT compares Beat the Kick to CAU, evaluating its effectiveness in enhancing motivation for change and stimulating treatment engagement. A major strength of the study lies in its robust design, including individual-level randomization through a Zelen design [37], the use of validated instruments such as the TSRQ [21, 26] and the BPNSFS-ID [34], and comprehensive follow-up assessments.

Previous studies have shown that individuals with MID frequently experience low levels of treatment readiness, due to factors such as poor insight into their substance use, limited verbal skills, and challenges in expressing personal goals [10, 11]. These factors not only limit engagement but also contribute to exclusion from traditional addiction services [6, 9]. The current study builds on prior work demonstrating that motivation is a key predictor of treatment adherence and success across populations [12], and that supporting autonomy, competence, and relatedness enhances this motivation in people with intellectual disabilities [13, 26].

The structure and content of Beat the Kick incorporate several of these principles. The intervention integrates techniques from motivational interviewing and cognitive-behavioral therapy, adapted for individuals with MID through simplified language, visual support, and personalization [13, 16–18, 21]. The inclusion of trusted caregivers and the focus on real-life examples are consistent with calls for concrete, individualized approaches that acknowledge the cognitive and communicative characteristics of this population [11, 19].

Importantly, the focus on pre-treatment motivation reflects growing recognition that many individuals with MID and substance abuse issues fall into a treatment gap—not yet motivated or eligible for intensive addiction treatment, yet clearly in need of support [8, 27]. Previous research on the earlier version of Beat the Kick showed promising outcomes using a single-case experimental design [21], but generalizability remained limited. The current RCT responds to this limitation by testing the intervention across six care organizations, enhancing external validity.

Nonetheless, several limitations must be considered. Due to the nature of the intervention, neither participants nor facilitators could be blinded to group assignment, which increases the risk of performance bias [37]. Additionally, reliance on self-report measures for substance use and psychological needs may introduce social desirability bias—particularly relevant in MID populations, who are known to provide socially desirable answers [13]. However, the instruments used in this study have been validated in this population and were supported with visual aids to facilitate comprehension and reduce misunderstanding [23, 35, 39].

Another important consideration concerns participant dropout, which is a known challenge in studies involving individuals with co-occurring MID and substance abuse problems [1, 10, 28]. Despite proactive strategies to support retention—such as flexible scheduling and involvement of caregivers—a certain level of dropout is expected. Beyond its quantitative impact on statistical power, dropout may also introduce systematic bias, particularly if individuals who discontinue differ meaningfully from those who remain in the study. For instance, participants with lower verbal ability, greater psychological distress, or more unstable living conditions may be more likely to disengage, thereby skewing the sample towards individuals with more stable or supportive environments.

To better understand this dynamic, the study will qualitatively document reasons for discontinuation, capturing both participant-reported factors (e.g., emotional burden, lack of perceived relevance) and contextual elements (e.g., caregiver availability, competing life stressors). This information will not only support transparency in reporting but also inform interpretations of the intervention’s reach and generalizability. Furthermore, identifying patterns among dropouts may help illuminate barriers that future adaptations of the intervention need to address. In this sense, dropout is not merely a methodological limitation but also a source of insight into motivational and contextual vulnerabilities within the target population.

While the current study compares the intervention to standard care, future research may benefit from including an active control group to further isolate mechanisms of change. Moreover, adaptations of Beat the Kick could be explored for people with more severe disabilities or psychiatric comorbidities, such as those with more profound cognitive impairments or complex trauma histories [11, 15, 22].

By focusing on intrinsic motivation and supporting basic psychological needs, this study contributes to a growing body of evidence suggesting that motivation is not only a predictor of outcomes, but also a key starting point in treatment for people with MID and substance abuse problems [12, 13, 26]. If proven effective, Beat the Kick has the potential to fill a critical gap in addiction care, offering a low-threshold, evidence-based approach that can be implemented within a wide range of intellectual disability services.

## Trial status

Protocol version 1.0, dated 11 June 2025. Recruitment is expected to start in July 2025 and is anticipated to be completed by June 2026.

## Supplementary Information

Additional file 1

Additional file 2

Additional file 3

## Data Availability

Researchers on the sponsor’s research team have access to the dataset. Additional data needed to support the protocol can be provided upon request to other members of the research team and regulatory authorities listed in the information letter.

## Abbreviations

BPNSFS-ID: Basic Psychological Need Satisfaction and Frustration Scale – Intellectual Disability
CAU: Care As Usual
MID: Mild intellectual disabilities
TSRQ: Treatment Self-Regulation Questionnaire
