# Supplementary material for: Effectiveness of the Beat the Kick intervention in addressing substance abuse among adults with mild intellectual disabilities in the Netherlands: study protocol for an open-label, multicenter, superiority randomized controlled trial
Source: Trials. 2025 Nov 27;26:597. doi: 10.1186/s13063-025-09299-3 (PMC12751549; doi:10.1186/s13063-025-09299-3)
Supplement: Supplementary file 2 — Additional file 2 [file 13063_2025_9299_MOESM2_ESM.pdf]

Informatiebrief voor de experimentele groep: Deelname aan het programma “Sterker dan de Kick”

Beste lezer,

Wij doen onderzoek vanuit de Universiteit van Tilburg. We willen je vragen of je mee wilt doen aan ons onderzoek. Het onderzoek heet “Sterker dan de Kick” en richt zich op de behandeling van middelengebruik bij volwassenen met een licht verstandelijke beperking. Dit onderzoek gaat over jouw gebruik van alcohol of cannabis. We willen graag onderzoeken of deze training echt werkt in het verminderen van deze gewoontes.

Waarom doen we dit onderzoek? Soms hebben mensen gewoontes, zoals het drinken van alcohol of het gebruiken van drugs, die niet goed zijn voor hun gezondheid. Dit geldt ook voor volwassenen met een lichte verstandelijke beperking. Met dit onderzoek willen we ontdekken of de training ‘Sterker dan de Kick’ helpt om deze gewoontes te verminderen. Door mee te doen, help je ons om deze training beter te maken en de ondersteuning voor jou en anderen in vergelijkbare situaties te verbeteren.

Wat houdt deelname in? Je doet mee aan het programma “Sterker dan de Kick.” Dit programma bestaat uit 10 wekelijkse bijeenkomsten van ongeveer 45 tot 60 minuten. Tijdens deze bijeenkomsten leer je:

Hoe je situaties herkent waarin je meer zin krijgt in alcohol of drugs;

Hoe je gezondere keuzes kunt maken;

Hoe je hulp kunt vragen aan mensen om je heen.

Daarnaast vragen we je om vier keer een vragenlijst in te vullen, op de volgende momenten:

Voor de start van het programma – voordat je begint, zodat we een goed beeld krijgen van je motivatie en je huidige middelengebruik. Een onderzoeker zal erbij zijn om uitleg te geven en je te helpen als dat nodig is.

Na afloop van het programma – direct na de laatste bijeenkomst, om te zien wat je hebt geleerd en hoe je motivatie en middelengebruik zijn veranderd.

Ongeveer één maand na afloop – om te kijken hoe je het programma toepast in je dagelijks leven en of er veranderingen zijn in je motivatie en middelengebruik.

Ongeveer zes maanden na afloop – om te zien of de resultaten blijvend zijn en hoe het nu met je gaat.

Bij alle meetmomenten vinden de metingen plaats in overleg met de onderzoeker op locatie. De onderzoeker is beschikbaar om uitleg te geven en je te helpen bij het invullen van de vragenlijsten.

Wil je meedoen aan het onderzoek?

Vul dan het toestemmingsformulier op pagina 4 in en mail het ingevulde formulier naar R.Gideonse@tilburguniversity.edu.

Wat verder nog belangrijk is:

- Jij bent door loting in de groep gekomen die het programma **"Sterker dan de Kick"** volgt. De groepen zijn ingedeeld door het trekken van loten, zodat we eerlijk kunnen testen of dit programma beter werkt dan de standaard hulp, zoals begeleiding of gesprekken, die mensen normaal krijgen.
- In het programma "Sterker dan de Kick" leer je hoe je jouw middelengebruik kunt verminderen. Mensen in de andere groep krijgen de hulp die ze normaal zouden krijgen, zoals

gesprekken met een hulpverlener of begeleiding vanuit een zorginstelling. Door de twee groepen te vergelijken, ontdekken we of "Sterker dan de Kick" beter helpt dan deze gewone hulp.

- Alles wat je vertelt of wat we over jou verzamelen in het onderzoek, blijft vertrouwelijk. We zorgen ervoor dat jouw naam en persoonlijke informatie worden weggehaald als we de resultaten met anderen delen, zodat niemand kan zien dat de gegevens over jou gaan.
- Jouw gegevens worden opgeslagen op een beveiligde server van de universiteit, een speciale computer die goed beschermd is tegen ongewenste toegang. De gegevens blijven daar 15 jaar bewaard en alleen onderzoekers die aan dit project werken, kunnen ze bekijken.
- Voor dit onderzoek vragen we om jouw leeftijd, geslacht, IQ, en diagnoses van je behandelaar. Deze gegevens slaan we veilig op en delen we niet met anderen buiten het onderzoek.
- Jouw gegevens worden alleen gebruikt voor dit onderzoek. Voor mogelijk vervolgonderzoek of het delen met derden zullen wij jouw gegevens eerst pseudonimiseren. Dit betekent dat jouw naam en andere persoonlijke gegevens worden vervangen door een unieke code, bijvoorbeeld een combinatie van letters en cijfers die alleen binnen het onderzoek wordt gebruikt. Deze code zorgt ervoor dat de gegevens niet direct naar jou herleidbaar zijn. Een aparte lijst met de koppeling tussen deze code en jouw persoonlijke gegevens wordt veilig bewaard en is alleen toegankelijk voor de onderzoekers. Mochten we jouw niet-gepseudonimiseerde gegevens willen gebruiken, dan vragen we hiervoor apart jouw toestemming.
- Je mag op elk moment stoppen met het onderzoek, zonder dat je hoeft te zeggen waarom. Dit heeft geen invloed op de hulp die je ontvangt. Alles wat je hebt verteld of ingevuld, blijft vertrouwelijk. Dit betekent dat alleen de onderzoekers jouw gegevens kunnen zien en dat we ze niet met anderen delen.
- Je hebt het recht om de gegevens die over jou zijn verzameld in te zien of om deze te laten verwijderen als je dat wilt.
- Dit onderzoek is goedgekeurd door de Ethische Toetsingscommissie van Tilburg Universiteit (ERB). Een ethische toetsingscommissie is een groep experts die controleert of een onderzoek eerlijk en veilig is voor de deelnemers. "ERB" staat tussen haakjes omdat dit de afkorting is van de volledige naam en terugkomt in het e-mailadres waarmee je contact kunt opnemen. Als je vragen of klachten hebt over het onderzoek, kun je altijd contact opnemen met de Ethische Toetsingscommissie of dit bespreken met je behandelaar. Het mailadres is: ERB@tilburguniversity.edu

Heb je nog vragen? Die kun je altijd stellen aan één van de onderzoekers: Rosemarie Gideonse , +31134663530, R.Gideonse@tilburguniversity.edu

Alvast bedankt!

Met vriendelijke groet,

Onderzoeker Rosemarie Gideonse (Tilburg University)

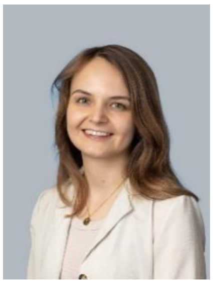

Ook namens:

Onderzoeker Noud Frielink (Tilburg University)

Onderzoeker Carlo Schuengel (Vrije Universiteit)

Onderzoek Joanneke van der Nagel (Universiteit van Twente)

Onderzoeker Petri Embregts (Tilburg University)

TOESTEMMINGSFORMULIER

Onderzoek: Sterker dan de kick – Interventie bij middelengebruik voor volwassenen met een licht verstandelijke beperking

Ik bevestig dat:

- Ik de informatie over het onderzoek heb gelezen.
- Ik vragen kon stellen aan de onderzoeker en deze zijn beantwoord.
- Ik vrijwillig deelneem aan het onderzoek.
- Ik weet dat ik op elk moment mag stoppen met het onderzoek. Dit heeft geen gevolgen en ik hoef geen reden te geven.

Toestemming

Ik geef toestemming voor:

- Deelname aan het onderzoek, dat bestaat uit tien wekelijkse sessies in het kader van het programma "Sterker dan de kick", met als doel te onderzoeken of deze behandeling echt werkt in vergelijking met de gebruikelijke zorg.
- Het verzamelen van informatie over mijn leeftijd, geslacht, IQ, en eventuele diagnoses bij mijn behandelaar, om inzicht te krijgen in mijn situatie en achtergrond.
- Het delen van informatie uit dit onderzoek met anderen gebeurt op een manier waarbij mijn gegevens eerst worden pseudonimiseerd. Dit betekent dat mijn naam wordt vervangen door een unieke code, zodat niemand kan zien dat de informatie over mij gaat.
- Het opslaan van de informatie uit dit onderzoek op een beveiligde schijf van de universiteit voor een periode van 15 jaar, zodat de gegevens vertrouwelijk en veilig blijven**.**

In te vullen door de deelnemer:

Naam deelnemer……………………………………………………………………………

Datum………………………………………………………………………………...

Handtekening deelnemer…………………………………………………………...

In te vullen door onderzoeker:

Ondergetekende verklaart dat de hierboven genoemde persoon zowel schriftelijk als mondeling over het bovenvermelde onderzoek is geïnformeerd. Hij/zij/die verklaart tevens dat een voortijdige beëindiging van de deelname door bovengenoemde persoon hier geen reden voor hoeft aan te geven.

Naam onderzoeker…………………………………………………………………………

Datum………………………………………………………………………………...

Handtekening onderzoeker…………………………………………………………...

De deelnemer ontvangt een kopie van de ondertekende toestemmingsverklaring
